# The diagnostic accuracy of ultrasound and genomic tests for the diagnosis of autosomal-dominant polycystic kidney disease: a systematic mapping review

**DOI:** 10.1093/ckj/sfaf187

**Published:** 2025-06-13

**Authors:** Sue Harnan, Matthew Gittus, Louise Falzon, Miranda Durkie, Olena Mandrik, Albert C Ong, James Fotheringham

**Affiliations:** Sheffield Centre for Health and Related Research, University of Sheffield, Sheffield, UK; Sheffield Centre for Health and Related Research, University of Sheffield, Sheffield, UK; Sheffield Centre for Health and Related Research, University of Sheffield, Sheffield, UK; Sheffield Diagnostic Genetics Service, North East and Yorkshire Genomic Laboratory Hub, Sheffield Children's NHS Foundation Trust, Sheffield, UK; Sheffield Centre for Health and Related Research, University of Sheffield, Sheffield, UK; Division of Clinical Medicine, University of Sheffield and Sheffield Kidney Institute, Sheffield, UK; Sheffield Kidney Institute, University of Sheffield, Sheffield, UK

**Keywords:** ADPKD, detection rate, diagnosis, genomic tests, ultrasonography

## Abstract

**Background:**

Genomic and ultrasound tests can provide diagnostic and prognostic information on autosomal-dominant polycystic kidney disease (ADPKD), and can screen first-degree relatives in whom early diagnosis can be advantageous. We conducted a systematic mapping review on test accuracy and characteristics over time.

**Methods:**

Medline, Embase, and Cochrane were searched (August 2023) for studies in first**-**degree relatives/individuals clinically diagnosed with ADPKD receiving genomic or ultrasound tests. Acceptable reference standards for sensitivity/detection rate and specificity were definitive imaging or genomic confirmation. Genomic studies were categorized by technology and read length. Relationships between sensitivity, specificity, genomic technology, diagnostic criteria/reference standard, and genes tested were compared.

**Results:**

From 1029 non-duplicate titles retrieved, 51 genomic and 7 ultrasound studies were included. There were no genomic studies in first-degree relatives. Among studies in patients with clinical diagnoses, genomic sequencing methodologies were highly heterogeneous [next generation (short read (*n* = 20), long read (*n* = 1)), targeted Sanger (*n* = 19), whole exome (*n* = 1) with additional multi-ligation probe analysis (*n* = 13)]. Median sensitivity was 78% (Interquartile range 65% to 88%). Ultrasound sensitivity and specificity generally improved with age and were worse in *PKD2* patients compared to *PKD1* (lowest reported 31% and 88%, respectively, in polycystic kidney disease (*PKD*) 2 patients aged 5–14; highest 100% and 100%, respectively, in multiple gene/age categories).

**Conclusions:**

Despite technological advances, sensitivity of genomic tests appeared static between 2000 and 2023. Possible explanations include clinical diagnostic criteria (and hence populations recruited) widening from *PKD1* to include *PKD2* and atypical phenotypes, and small incremental gains of testing genes other than PKD1 and PKD2. For people at risk of ADPKD in genetically unresolved families, the accuracy of ultrasound is uncertain. Unified genomic test taxonomies would facilitate future reviews.

**Registration:** PROSPERO CRD42023456727.

## Graphical Abstract

**Graphical Abstract:**
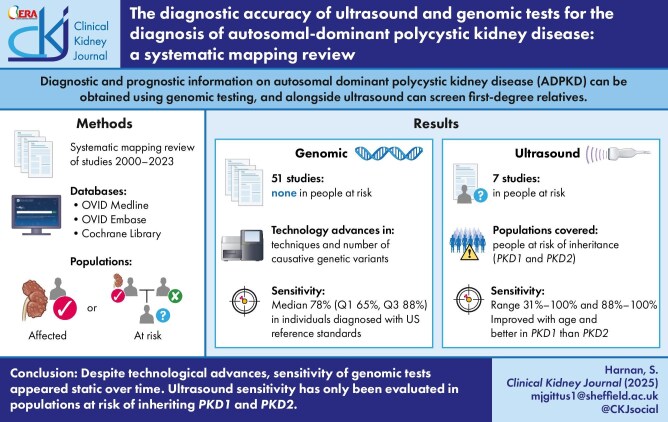

KEY LEARNING POINTS **What was known**:

- People at risk of inheriting ADPKD can be screened for the disease using ultrasound or genomic testing.
- Genomic testing technologies have evolved rapidly over the past 20 years.
- With the cost of genomic testing falling, some clinicians now have the choice between the two screening methods when the genomic variant in the affected individual is known.

**This study adds:**

- We found no genomic studies conducted in patients at risk of ADPKD, only in those already diagnosed using other methods.
- Overall, despite improvements in genomic methods, the sensitivity of genomic tests reported in the studies does not appear to have improved over time; this may be due to the widening population being tested and small incremental gains in detection rate provided by testing genes other than PKD1 and PKD2.
- We found no studies evaluating ultrasound in patients with genetic variants other than PKD1 and PKD2. Ultrasound test accuracy therefore remains unclear in these growing populations.

**Potential impact:**

- Clinicians screening populations at risk of ADPKD should appreciate that the sensitivity and specificity of ultrasound in relatives of those with ADPKD not caused by PKD1 and PKD2 is unknown.
- Decision-makers considering investing in genomic technologies should be aware of the relatively small incremental value of broader genomic panels when individuals affected by these variants are few.

## INTRODUCTION

Autosomal-dominant polycystic kidney disease (ADPKD) is the most common hereditary kidney disease, affecting an estimated 12 million individuals worldwide [1]. Being dominantly inherited, first-degree relatives have a 50% risk of developing the condition [2]. It is characterized by cystic expansion of the kidneys, progressing to bilateral kidney enlargement and subsequent chronic kidney disease (CKD) [3]. Symptoms typically begin around age 30 [4], and 50% of people with ADPKD require kidney replacement therapy by age 60 [5]. Although ADPKD is primarily caused by variants in *PKD1* and *PKD2* genes, ongoing discoveries of other causative genes have revealed greater genomic heterogeneity than previously understood [3, 6]. Even within *PKD1* and *PKD2* genes, there is significant allelic heterogeneity with >1200 and almost 190 pathogenic/likely pathogenic variants identified for *PKD1* and *PKD2*, respectively [7]. Most identified families have unique variants, with fewer than 2% of unrelated ADPKD-affected families sharing the same variant [8].

ADPKD diagnosis is mostly based on imaging and family history, and it can be difficult to differentiate from other cystic kidney diseases when imaging results are atypical or in young individuals with a negative family history [6]. By age 40, a diagnosis of ADPKD can be ruled out in people who have no more than one kidney cyst [9]. Genomic testing can provide a definitive diagnosis for patients, relatives at risk of inheriting the disease, and for individuals who are seeking genomic consultation prior to pre-implantation genomic diagnosis for reproduction or living kidney donor transplantation [6]. If possible, genomic testing of a family member who has a clinical diagnosis of ADPKD using a full diagnostic genomic test, usually including *PKD1* and *PKD2* genes as a minimum, is the recommended first step when genomic testing individuals at risk of inheriting ADPKD is being performed. If a pathogenic variant is identified in this family member, then predictive testing in their relatives can be offered by targeted analysis of the familial pathogenic variant.

Historically, guidelines have hesitated to recommend genomic screening due to costs and limited accessibility [10]. The Kidney Disease Improving Global Outcomes (KDIGO) clinical practice guidelines state that an ultrasound diagnosis can be used even when the family is genetically resolved [11]. These guidelines have been designed to be applicable to healthcare systems worldwide and as costs associated with genomic tests drop, gene panels broaden, and technology advances, a review of contemporary evidence to inform clinical practice guidelines is required. Earlier diagnosis has the potential to enable earlier management and improve outcomes for people with ADPKD. This can occur through earlier access to lifestyle and medication interventions, family planning, and living donation information [12, 13]. This systematic mapping review aims to describe and characterize the available diagnostic accuracy literature relating to ultrasound and genomic tests for people at risk of ADPKD. We aim to look at the changes in technology and chart the sensitivity of genomic tests over time and the diagnostic accuracy of ultrasound tests, to provide an overview of this fast-paced and complex topic.

## MATERIALS AND METHODS

This systematic mapping review is reported in line with recommendations made by PRISMA for scoping reviews [14], since there is no guidance for mapping reviews. We also considered relevant items from the PRISMA guidance for reporting diagnostic test accuracy reviews [15]. There is no standard definition of a mapping review [16], but they are generally descriptive in nature, do not include statistical synthesis, but rather use graphical, tabular, and narrative methodologies to characterize the literature.

The protocol was registered on the PROSPERO database (record number CRD42023456727), but some changes were made to the protocol as detailed in Online Supplement 1

### Search strategy

Potentially relevant articles were identified by searching Ovid Medline, Ovid Embase, and the Cochrane Library from inception to August 2023. Relevant subject headings and free-text terms to represent ‘Autosomal Dominant Polycystic Kidney Disease’ AND ‘ultrasound’ OR ‘genetic screening’ were used. A validated search filter to identify diagnostic studies was applied [17], but the studies were not limited by year or language. Reference lists of relevant studies and reviews, and relevant articles in the Similar Articles feature in PubMed, and the Cited Reference Search in ISI Web of Science were also screened. The following relevant conferences were searched for the past 3 years: American Society of Nephrology Kidney Week, World Congress of Nephrology, and European Renal Association Congress. Full details of the search dates and strategies are available in Online Supplement 2

### Study selection

The selection criteria for the review are reported in Table 1. Studies of ultrasound were included if they recruited first-degree relatives of people with ADPKD (i.e. people with 50% risk of having ADPKD) and the reference standard was imaging after age 40 years according to published criteria (e.g. Pei *et al.* [18], Pei *et al.* [19], Torres *et al.* [20]), or genomic confirmation by any genomic method (e.g. gene linkage analysis, Sanger sequencing). Studies using high-resolution ultrasound were excluded because standard ultrasound remains the predominant method in clinical use.

**Table 1:** The selection criteria for the review.

	Genomic test or strategy utilizing genomic test(s)		Grey-scale ultrasound
Population	1st degree relatives of patients with an ADPKD diagnosis (clinical or genomic)	People with a clinical ADPKD diagnosis according to Pei *et al.*, 2009 [18] or Ravine *et al.* 1994 [28].^a^ With or without a family history, related or unrelated (preferred).^c^	1st degree relatives of patients with an ADPKD diagnosis (clinical or genomic) Studies in foetuses were excluded
Index test	Genomic test or diagnostic strategy utilizing genomic tests		Grey-scale ultrasound
Reference standard	Imaging according to Pei *et al.* 2009 [18] or Ravine *et al.* 1994 [28].^a^ or genomic confirmation^b^		Imaging according to Pei *et al.* 2009 [18] or Ravine *et al.* 1994 [28],^a^ after age 40 or genomic confirmation^b^
Target condition	ADPKD		
Outcome	● Sensitivity and specificity; TP, FP, TN, FN. If not available, diagnostic rate (sensitivity)^d^. ● Rates of pathogenic variants, variants of unknown significance, no pathogenic variants etc.		
Study design	Diagnostic test accuracy studies. If none available, studies reporting sensitivity only were eligible.		

Abbreviations: CT, computed tomography; FN, false negative; FP, false positive; MRI, magnetic resonance imaging; TN, true negative; TP, true positive.

a Studies that used alternative criteria, e.g. Torres *et al.* 2012 [20], for atypical presentations were also included; Studies that considered negative scans in patients before 40 years of age as definitive were excluded; studies that applied Pei *et al.* 2015 [19] criteria for CT and MRI imaging or Pei *et al.* 2009 [18] or Ravine *et al.* 1994 [28] criteria for ultrasound were included. In cases of doubt about recruitment criteria, clinical experts were consulted.

b No limits were placed on the type of genomic confirmation. For genomic studies, only extracted data using a genomic reference standard if no data using an imaging reference standard were available from that study.

c Where there was a choice, data for unrelated probands were extracted in preference to data for a mix of related and unrelated participants.

d Diagnostic rate was only acceptable where studies recruited only patients with a clinical ADPKD diagnosis, and in this circumstance is equivalent to sensitivity of the test since all participants have clinical ADPKD (i.e. are reference-standard positive and therefore comprise all true positives and false negatives, but no true negatives or false positives).

Studies of genomic tests or diagnostic strategies including genomic tests were included if they recruited either first-degree relatives of people with an ADPKD diagnosis, or people with or without a family history with a clinical ADPKD diagnosis according to published diagnostic criteria (e.g. Pei *et al.* [18], Pei *et al.* [19], Torres *et al.* [20]), because these are the groups the tests would be used in. The reference standard could be a diagnosis using published criteria, or a genomic diagnosis. This was a change from the published protocol because no studies met the original criterion (see Online Supplement 1).

In both reviews, prenatal populations were excluded since short follow-up meant it was not clear if all the foetuses grew up to have the disease, and the pathogenic variant may have resulted in prenatal death such that testing in a child would never have been necessary.

We did not restrict inclusion to studies using the American College of Medical Genetics and Genomics guidance for the interpretation of sequence variants [21], but attempted to standardize definitions where possible (see the section on ‘Sensitivity’).

Two reviewers (S.H. and M.G.) separately used Covidence with AI-assisted study prioritization to screen studies according to the inclusion criteria, considering first the title and abstract, then examining the full texts of the remaining articles. Any disagreements were resolved through discussion and involvement of a third reviewer (J.F.).

### Data extraction and quality assessment

A data extraction form was created in Google Sheets, piloted on two articles and improved where necessary. Data extraction fields and methods are provided in Online Supplement 2 but briefly comprised data extraction, data coding, and data double-checking by a second reviewer with resolution of disagreements through discussion.

As none of the studies of genomic tests were true diagnostic test accuracy studies and were therefore of generally low quality, QUADAS 2 [22] quality assessment was not performed.

### Mapping analysis

The evidence map was primarily analysed according to two main criteria:

(i) Test type: ultrasound studies were grouped separately from genomic studies. Genomic studies were then categorized according to the sequencing technology used. These sequencing technology components are defined in Table 2 and categories described in Online Supplement 3 Studies were grouped by technology used (Sanger or next generation), the genomic target (targeted gene, whole exome or whole genome), and whether the read length was short (first and second generation) or long (third generation).
(ii) Population: the criteria used to recruit patients may affect the detection rate since early clinical definitions were largely based on *PKD1* (Ravine [28]) and then expanded to *PKD2* (Pei [18] and Pei [19]). Studies were therefore grouped according to the criteria used to define the clinical diagnosis of ADPKD. Clinical criteria included Ravine [28], Pei [18] for ultrasound, and Pei [19] for MRI or sometimes CT. Other criteria could be used for atypical presentations, such as Torres *et al.* [20]. Studies could cite published criteria, or accurately describe the criteria that were then matched to the corresponding citation. Studies that recruited patients according to a genomic diagnosis were grouped separately.

**Table 2:** Sequencing technology component definitions [81, 82].

Components of genomic testing	Definition
Technology	The specific tools and platforms used to sequence and analyse DNA
Read length	The length of DNA sequence read by a sequencing machine in a single run, typically ranging from 50 to several thousand base pairs
Enrichment method	Techniques used to selectively capture and sequence specific regions of the genome
Analysis	Computational processes and algorithms used to interpret raw sequencing data including the examination of specific sets of genes
Genomic structural variation analysis	The identification of changes such as deletions, insertions, inversions, translocations, single nucleotide variations, and copy number variations

Several plots were then generated using R version 4.4 to show trends over time for factors including recruitment criteria, test types, gene targets, and detection rate. Changes in longitudinal detection rate were estimated using the ggplot2 generalized linear model smoothed conditional mean function, weighted for study size, with a binomial link function.

## RESULTS

**Figure 1:**
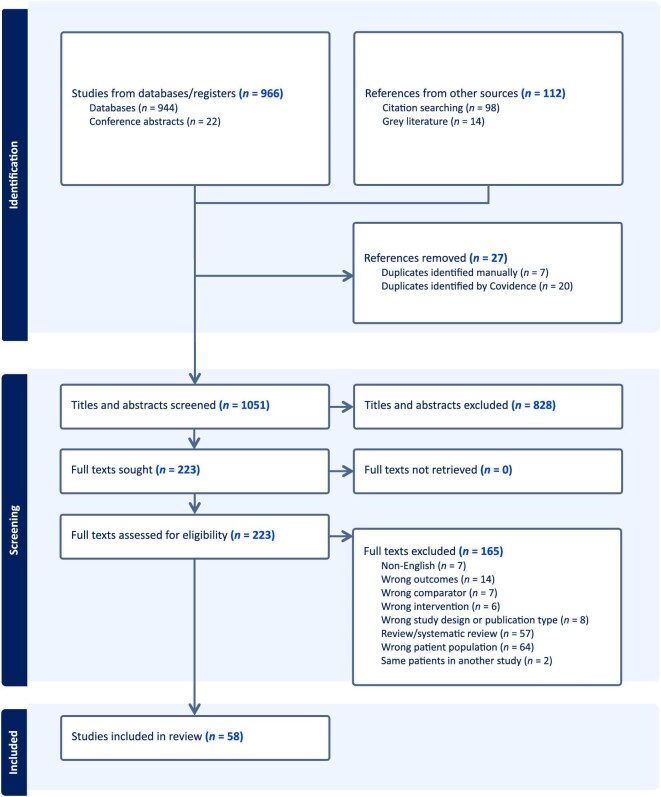
PRISMA flow chart showing the process of study selection for the review.

The search strategy retrieved a total of 1078 titles, from which 27 duplicates were removed. Of the 1051 records remaining, 828 were excluded on the basis of their title or abstract. The full text of 223 studies were assessed for eligibility, and of these 165 were excluded (see Fig. 1 for reasons). Seven studies [18, 23–28] of ultrasound and 50 studies [6, 8, 29–76] of genomic tests were included in the review.

### Studies of genomic tests

#### Location of studies

**Figure 2:**
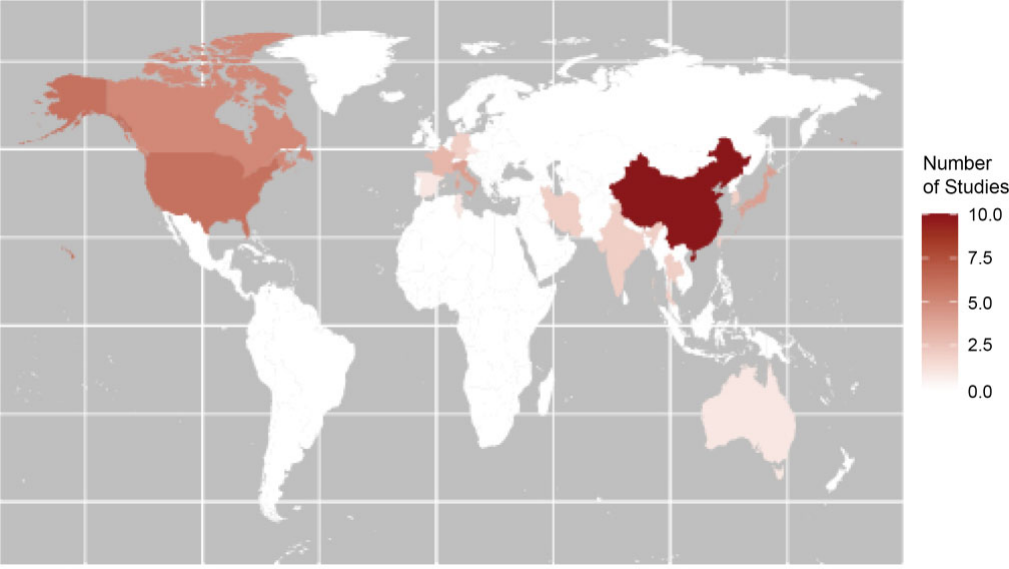
Map of origin of included studies.

The countries of origin of the included studies are mapped in Fig. 2. The country contributing the most studies was China (*n* = 10) [42, 46, 49, 51, 52, 71–75], followed by the USA (*n* = 6) [35, 63, 64, 67, 68, 77]. The remainder were from across the globe, including Canadian, European Middle Eastern, and Asian studies.

#### Recruitment criteria

**Figure 3:**
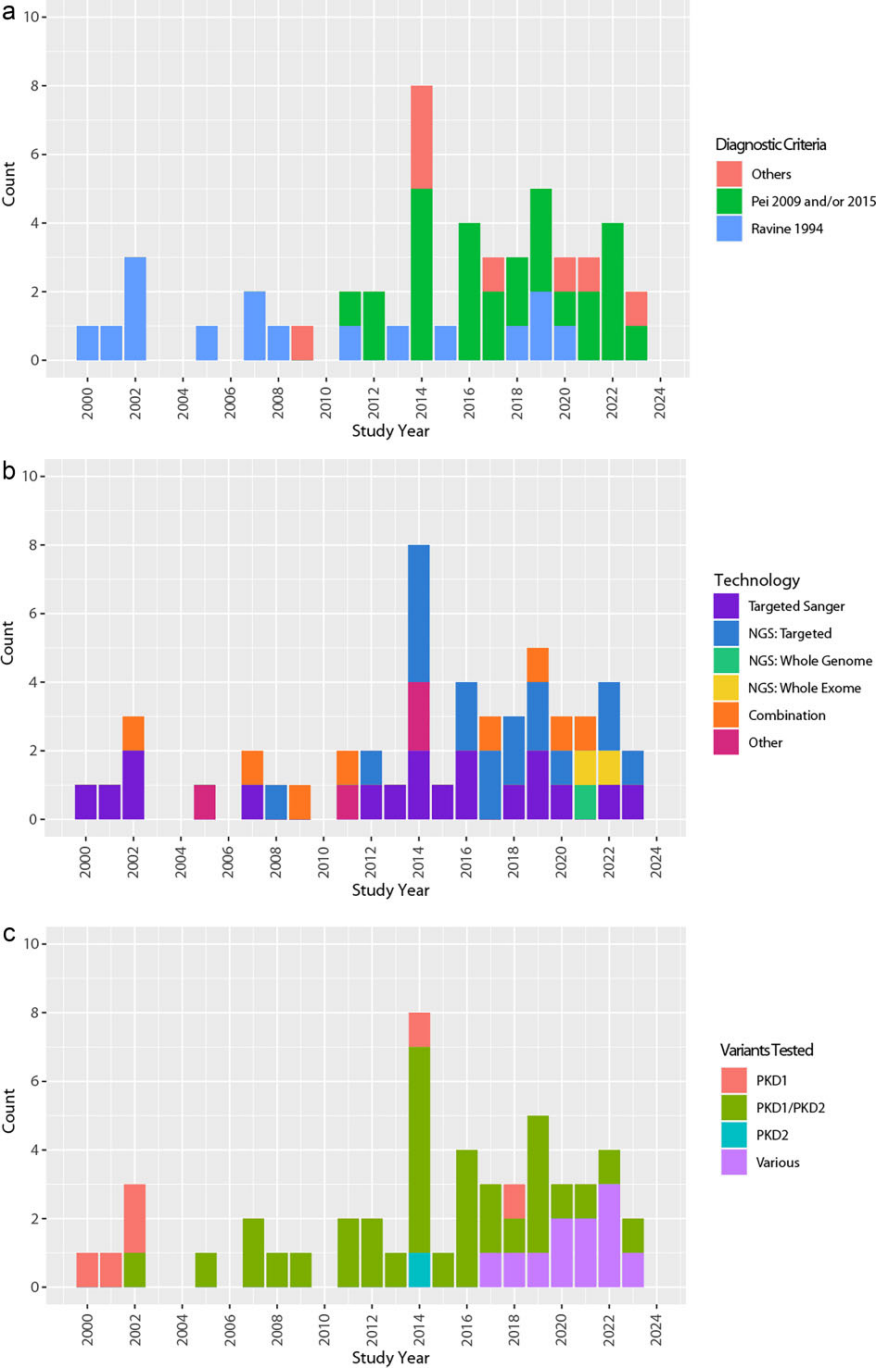
Charts of study characteristics over time. (**a**) Diagnostic criteria/radiological reference standard for inclusion of individuals with clinical diagnosis of ADPKD by year of study publication; (**b**) genomic test technology by year of study publication; and (**c**) genes analysed by genomic tests by year of study publication.

Among the 51 genomic test studies (Table 3) patients were recruited according to Pei *et al.* 2009 [18] and its extension Pei *et al.* 2015 [19] (*n* = 25 studies) [6, 8, 30, 31, 34, 35, 37, 38, 41, 43, 44, 46–49, 53, 55–57, 62, 65–67, 71, 72], most often. These criteria were derived in *PKD1* and *PKD2* patients. Ravine *et al.*’s [28] criteria, which targeted *PKD1* patients, were used in 16 studies [29, 33, 36, 40, 45, 51, 58–61, 63, 64, 73–76]. Other imaging criteria (Torres *et al.* [20], Torres *et al.* 2017 [78], KDIGO guideline criteria) were used in a further five studies [39, 42, 50, 52, 54], and these are likely to recruit a wider population than just *PKD1* and *PKD2*. Four studies recruited people using genomic tests: one [69] targeted people with *PKD1* and *PKD2* pathogenic variants and aimed to include as many different variants as possible, while the other three [32, 68, 77] did not state which genes were targeted. One study used *PKD2* families previously analysed by linkage analysis [70]. Surprisingly, Ravine *et al*.’s 1994 [28] criteria were used to recruit patients in four studies [29, 58, 61, 74] published between 2018 to 2020. However, overall, due to the criteria used, the populations included in more recent studies were more heterogeneous and less phenotypically characteristic of *PKD1*/*PKD2* pathogenic variants (Fig. 3a).

**Table 3:** Characteristics of studies included in the review.

Author, year, country	*N* in study	Proband/per family?	Family history	Reference standard^a^	Genes targeted	Enrichment method	Small sequence variant analysis	Copy number variant analysis
**Targeted Sanger sequencing (*n* = 18)**								
Pei 2009 recruitment criteria (*n* = 4)								
Ali 2019 [30] Kuwait	6	Proband or per family	With family history	Pei 2009	*PKD1; PKD2*	Amplicon	NA	None
Audrezet 2012 [8] France	528 (with FH) 172 (Without FH)	Proband or per family	With family history Without family history	Pei 2009	*PKD1; PKD2*	Amplicon	NA	None
Hwang 2016 [43] Canada	220	Proband or per family	Either with or without family history	Pei 2009	*PKD1; PKD2*	Amplicon	NA	MLPA
Li 2022 [49] China	19	Proband or per family	Either with or without family history	Pei 2009	*PKD1; PKD2; GANAB*	Amplicon	NA	MLPA
Pei 2009 and Torres 2012 recruitment criteria (*n* = 1)								
Liu 2014a [52] China	10	Proband or per family	Either with or without family history	Family history: Pei 2009; no family history, Torres 2012	*PKD1*	Amplicon	NA	MLPA
Pei 2009/2015 recruitment criteria (*n* = 2)								
Carrera 2016 [34] Italy	440	Proband or per family	Either with or without family history	Pei 2009/2015	*PKD1; PKD2*	Amplicon	NA	MLPA
Orisio 2023 [57] Italy	198	Proband or per family	Either with or without family history	Pei 2009/2015	*PKD1; PKD2*	Amplicon	NA	MLPA
Ravine 1994 recruitment criteria (*n* = 10)								
Abdelwahed 2018 [29] Tunisia	18	Some related	Either with or without family history	Ravine 1994	*PKD1*	Amplicon	NA	MLPA
Burtey 2002 [33] France		Proband or per family	Either with or without family history	Criteria equivalent to Ravine 1994	*PKD1*	Amplicon	NA	None
Chang 2013 [36] Taiwan	46	Proband or per family	Either with or without family history	Ravine 1994	*PKD1; PKD2*	Amplicon	NA	MLPA
Garcia-Gonzalez 2007 [40] Canada	82	Proband or per family	Either with or without family history	Ravine 1994	*PKD1; PKD2*	Amplicon	NA	None
Inoue 2002 [45] Japan	8	Proband or per family	Not reported	Ravine 1994	*PKD1*	Amplicon	NA	None
Liu 2015 [51] China	49	Proband or per family	Either with or without family history	Ravine 1994	*PKD1; PKD2*	Amplicon	NA	MLPA
Pandita 2019 [58] India	125	Proband or per family	Either with or without family history	Ravine 1994	*PKD1; PKD2*	Amplicon	NA	MLPA
Phakdeekitcharoen 2000 [59] Thailand	37	Proband or per family	Not reported	Ravine 1994	*PKD1*	Amplicon	NA	None
Phakdeekitcharoen 2001 [60] Thailand	37	Proband or per family	Not reported	Ravine 1994	*PKD1*	Amplicon	NA	None
Raj 2020 [61] India	84	Proband or per family	Either with or without family history	Ravine 1994	*PKD1; PKD2*	Amplicon	NA	None
Genomic recruitment criteria (*n* = 1)								
Liu 2014b [77] USA	8	Proband or per family	Not reported	Sanger genotyping	PKD1; PKD2	Amplicon	NA	None
**Next-generation sequencing: targeted—short read (*n* = 17)**								
Pei 2009 recruitment criteria (*n* = 10)								
Choi 2014 [37] South Korea	20	Proband or per family	Either with or without family history	Pei 2009	*PKD1; PKD2*	Hybridization	Targeted panel	MLPA
Jin 2016 [46] China	148	Unclear	Either with or without family history	Pei 2009	*PKD1; PKD2*	Hybridization	Targeted panel	None
Kinoshita 2016 [48] Japan	101	Proband or per family	Not reported	Pei 2009	*PKD1; PKD2*	Amplicon	Targeted panel	MLPA
Mochizuki 2019 [55] Japan	111	Unclear	Not reported	Pei 2009	*PKD1; PKD2*	Hybridization + Amplicon	Targeted panel	MLPA
Ranjzad 2017 [62] Iran	18	Proband or per family	With family history	Pei 2009	*PKD1; PKD2*	Hybridization	Targeted panel	None
Rossetti 2012 [65] NR (possibly USA)	183	Proband or per family	Not reported	Pei 2009	*PKD1; PKD2*	Amplicon	Targeted panel	None
Tan 2014 [67] USA	25; 3; 25^b^	Unclear	Not reported	Sanger sequencing	*PKD1; PKD2*	Amplicon	Targeted panel	None
Xu 2018 [71] China	120	Proband or per family	Not reported	Pei 2009	*PKD1; PKD2*	Amplicon	Targeted panel	MLPA
Yang 2014 [72] China	7	Proband or per family	Either with or without family history	Pei 2009	*PKD1; PKD2*	Amplicon	Targeted panel	None
Yu 2022 [6] Taiwan	882	Proband or per family	Either with or without family history	Pei 2009	*PKD1, PKD2, PKHD1, GANAB, ALG8, DNAJB11*	Amplicon	Targeted panel	None
Pei 2015 recruitment criteria (*n* = 1)								
Hosseinpour 2022 [41] Iran	32	Proband or per family	Either with or without family history	Pei 2015	*PKD1; PKD2*	Hybridization	Targeted panel	None
Ravine 1994 recruitment criteria (*n* = 2)								
Zhang 2019 [74] China	62	Proband or per family	Not reported	Ravine 1994	*PKD1; PKD2*; PKHD1	Hybridization	Targeted panel	MLPA
Zhao 2008 [76] Canada	3	Proband or per family	Either with or without family history	Ravine 1994 in Probands	*PKD1; PKD2*	Amplicon	Targeted panel	None
Pei 2009/Torres recruitment criteria (*n* = 1)								
Fujimaru 2018 [39] Japan	53	Proband or per family	Without family history	CT or MRI (>10 cysts in each kidney), Pei 2009, Torres 2012, Torres 2017	69 genes causing hereditary renal cystic disease	Hybridization	Targeted panel	NGS CNV
Other imaging recruitment criteria^c^ (*n* = 2)								
Lindemann 2023 [50] Germany	441 123	Unclear	Either with or without family history	Imaging as per footnote^c^	*PKD1; PKD2; GANAB; HNF1b*	Amplicon	Targeted panel	None
Mantovani 2020 [54] Italy	191	Unclear	Either with or without family history	Pei 2009 criteria for US, MRI, CT if equivocal	*PKD1; PKD2*; 14 additional cystogenes (if negative)	Amplicon	Targeted panel	MLPA
Genomic criteria (*PKD1* or *PKD2* pathogenic variants) (*n* = 1)								
Trujillano 2014 [69] Spain	36	Unclear	Not reported	Sanger sequencing of *PKD1*/2 exons, and if negative MLPA	*PKD1; PKD2*	Amplicon	Targeted panel	None
**Next-generation sequencing: targeted—long read (*n* = 1)**								
Genomic recruitment criteria (*n* = 1)								
Borras 2017 [32] Europe	19	Proband/per family	NR	Genomic (short read WGS or WES)	*PKD1; PKD2*	SMRT	Panel	NGS breakpoint detection, MLPA
**Next-generation sequencing: WGS—short read (*n* = 1)**								
Pei 2009 recruitment criteria (*n* = 1)								
Mallawaarachchi 2021 [53] Australia	42	Proband/per family	Either with or without family history	Pei 2009	*PKD1; PKD2*	Hybridization	Virtual panel	NGS CNV, MLPA
**Next-generation sequencing: WES—short read (*n* = 2)**								
Pei 2009 and wider atypical disease recruitment criteria (*n* = 2)								
Chang 2022 [35] USA	235	Unclear	Either with or without family history	Pei 2009	*PKD1, PKD2*, and 11 atypical cystic genes (*ALG8, ALG9, DNAJB11, GANAB, HNF1B, IFT140, LRP5, PKHD, PRKCSH, SEC61B, SEC63)*	Hybridization	Virtual panel	NGS CNV
Elliott 2021 [38] Canada	18	Proband or per family	Either with or without family history	Typical ADPKD: Pei 2009 Atypical ADPKD: atypical kidney imaging (Mayo Class 2), no family history, atypical clinical presentation, suspicion for another genomic CKD	Typical ADPKD: *PKD1; PKD2* Atypical ADPKD: *PKD1, PKD2, COL4A1, DNAJB11, GANAB, HNF1B, REN, and UMOD.*	Hybridization	Virtual panel	None
**Tests in combination (*n* = 8)**								
DHPLC then first generation: targeted Sanger sequencing (*n* = 4)								
Ravine 1994 recruitment criteria (*n* = 3)								
Rossetti 2002 [63] USA	45	Proband or per family	Not reported	Ravine 1994 criteria	*PKD1; PKD2*	Amplicon	NA	None
Rossetti 2007 [64] USA (CRISP cohort)	127	Proband or per family	Not reported	Ravine 1994	*PKD1; PKD2*	Amplicon	NA	None
Yu 2011 [73] China	65	Proband or per family	Either with or without family history	Ravine 1994	*PKD1; PKD2*	Amplicon	NA	None
Genomic recruitment criteria (*n* = 1)								
Tan 2009 [68] USA	14	Unclear	Not reported	Unclear ‘PKD genotyping’ by reference lab	*PKD1, PKD2*	Hybridization	NA	None
**NGS targeted then Sanger (*n* = 1)**								
KDIGO guidelines (Chapman 2015) (*n* = 1)								
Hu 2021 [42] China	26	Proband or per family	Either with or without family history	KDIGO guidelines (Chapman 2015)	Tier 1: WES and *PKD1* Tier 2: *PKD1* (MLPA)	Hybridization	Targeted Panel/NA	MLPA
**NGS targeted then Sanger with MLPA; data also reported separately for NGS targeted then WES then MLPA (*n* = 1)**								
Pei 2009/2015 recruitment criteria (*n* = 1)								
Schonauer 2020 [66] Germany	100	Some related	Either with or without family history	Pei 2009 $\&$ Pei 2015	*PKD1, PKD2, GANAB, PKHD1, and HNF1B*	Hybridization	Targeted Panel/Virtual Panel	MLPA
**Sanger with MLPA then NGS targeted (*n* = 1)**								
Pei 2009/2015 recruitment criteria (*n* = 1)								
Iliuta 2017 [44] Not reported	205	Proband or per family	Either with or without family history (and reported separately)	Pei 2009 $\&$ Pei 2015	*PKD1; PKD2; GANAB; HNF1B*	Hybridization	NA/Targeted Panel	None
**Targeted NGS then Sanger with MLPA then familial segregation analysis (*n* = 1)**								
Pei 2009 recruitment criteria (*n* = 1)								
Kim 2019 [47] Korea	524	Proband or per family	Either with or without family history	Pei 2009	*PKD1; PKD2*	Hybridization	Targeted Panel/NA	MLPA
**Other types of test (*n* = 4)**								
HRM (*n* = 3)								
Pei 2009 recruitment criteria (*n* = 2)								
Bataille 2011 [31] France	37	Proband or per family	Not reported	Pei 2009	*PKD1; PKD2*	NA	NA	None
Obeidova 2014 [56] Czech republic	56	Proband or per family	Either with or without family history	Pei 2009	*PKD1; PKD2*	NA	NA	MLPA
PKD-2 linkage analysis recruitment criteria (*n* = 1)								
Virzi 2014 [70] Italy	16	Proband or per family	Not reported	*PKD2* linkage analysis	*PKD2*	NA	NA	None
**Single-strand conformation polymorphism analysis (*n* = 1)**								
Ravine 1994 recruitment criteria (*n* = 1)								
Zhang 2005 [75] China	24	Proband or per family	Not reported	Ravine 1994	*PKD1; PKD2*	NA	NA	None

a Reference standard: Terms such as ‘unified criteria’, ‘Ravine-Pei’, and so on were assumed to be Pei *et al.* 2009 [18].

b Three cohorts were reported: Patient with ADPKD previously analysed by Sanger sequencing (*n* = 25); ADPKD cases that tested negative by Sanger sequencing (*n* = 3); ADPKD not previously genomically tested (*n* = 25).

c Recruitment criteria: Lindeman *et al.* 2023 [50], if family history, Pei *et al.* 2009/2015 [18, 19], if no family history, at least 10 cysts per kidney, bilaterally enlarged kidneys, at least 1 classic extrarenal manifestation of ADPKD, and no extrarenal manifestations pointing toward differential diagnoses (e.g. hepatic fibrosis); Mantovani *et al.* 2020 [54], Pei *et al.* 2009 [18] for ultrasound, could use MRI or CT if equivocal.

#### Reference standard

In nearly all cases, the reference standard was the same as the recruitment criteria. As already noted, these studies are only able to estimate detection rate (sensitivity) and cannot estimate specificity.

#### Test types

Among the 51 genomic test studies [6, 8, 29–77] (Table 3), there was a similar number of studies of targeted Sanger sequencing (*n* = 18) [8, 29, 30, 33, 34, 36, 40, 43, 45, 49, 51, 52, 57–61, 77] and targeted short read next-generation sequencing (*n* = 17) [6, 37, 39, 41, 46, 48, 50, 54, 55, 62, 65, 67, 69, 71, 72, 74, 76]. There was only one study of targeted long read next-generation sequencing [32], one of WGS short read next-generation sequencing [53], two of WES short read next-generation sequencing [35, 38], eight tests used a combination of technologies [42, 44, 47, 63, 64, 66, 68, 73], and four reported on other types of genomic tests [31, 56, 70, 75]. Studies were published from 2000 to 2023 (date of searches).

Figure 3b charts the types of test used over time. Sanger sequencing has been used consistently throughout the period, while the application of next-generation technologies to ADPKD diagnosis was first reported in 2008 and use has increased over time. The one study of long read technology was published in 2017 [32]. Studies on tests used in combination started in 2002, with early studies focusing on DHPLC followed by Sanger sequencing [36, 63, 64, 68, 73], and later studies mostly using combinations of next-generation sequencing, MLPA and Sanger but not always in the same order [42, 44, 47, 66]. Other test types encountered included high-resolution melt (HRM) [31, 56, 70] and single-strand conformation polymorphism analysis (SSCP) [75].

#### Gene targets

The genes targeted by genomic tests also broadened over time (Fig. 3c). Four of the seven studies [29, 33, 42, 45, 52, 59, 60] that only focused on *PKD1* were among the five earliest studies conducted (2000 to 2002) [33, 45, 59, 60, 63]. Testing for genes beyond *PKD1* started with the inclusion of *PKD2* by Rossetti *et al*. [63], and expanded beyond *PKD1* and *PKD2* in 2017, when Iliuta *et al.* [44] included *GANAB* and *HNF1B*. Later tests [6, 38] broadened into *COL4A1, DNAJB11, REN*, and *UMOD*.

#### Sensitivity

**Figure 4:**
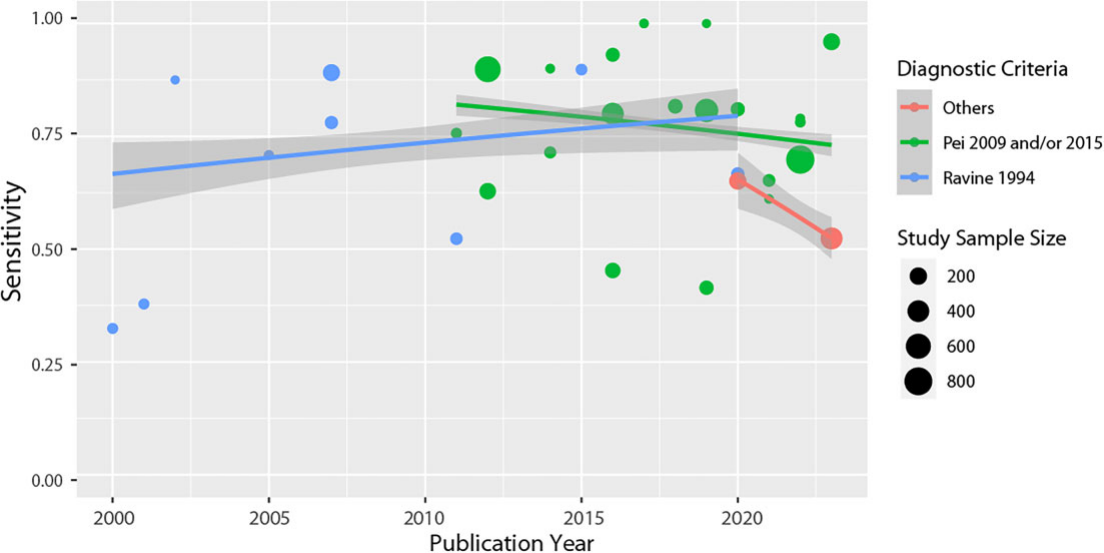
Diagnostic test accuracy (proportion with genomic variants classed as definitely pathogenic, pathogenic, likely pathogenic, and probability pathogenic or similar terms), stratified by genes targeted and recruitment criteria, by study publication year. Blue line, studies recruiting according to Ravine [28], with genomic testing for PKD1 or more; green line, studies recruiting according to Pei 2009/2015 [18, 19], with genomic testing for PKD1 and PKD2 or more; red line, studies recruiting according to other criteria, with genomic testing for more than PKD1 and PKD2. Studies weighted by size when estimating longitudinal changes and 95% confidence intervals (grey).

Heterogeneity in the terminology used to categorize pathogenic variants supported grouping terminology erring towards the variant being pathogenic together. e.g. pathogenic, probably/likely/definitely/strong pathogenic, disease-causative, possibly damaging. To plot detection rate over time a subgroup of studies that reported both pathogenic/definitely pathogenic and likely/probably pathogenic (or similar terms) variants were selected. Studies were further grouped into three categories, to match the recruitment criteria to the genes tested (Ravine [28] criteria, only genomic tests for *PKD1* or more were included; Pei 2009/2015 [19], genomic tests for *PKD1* and *PKD2* or more were included; other criteria, only genomic tests for *PKD1, PKD2*, and at least one other gene were included). Figure 4 plots the sensitivity of the tests for these three subgroups. Across all three groups, the median detection rate was 78% (interquartile range 65% to 88%, total range 32% to 100%). Sensitivity remained fairly stable over the years (Ravine [28] subgroup, range 32% [59] to 90% [51] and Pei 2009/2015 subgroup, range 41% [55] to 100%) [30, 62] or had too few points for inference (Others subgroup).

### Ultrasound studies

The characteristics of the seven studies [18, 23–28] are outlined in Table 4. The date of studies ranged from 1990 [27] to 2009 [18] (NB Pei *et al.* 2015 [19] did not meet the inclusion criteria as it used high-resolution ultrasound). All [18, 23–28] recruited people at 50% risk of ADPKD from families with. PKD1 (*n* = 4) [24, 25, 27, 28], PKD2 (*n* = 1) [23], or PKD1 and PKD2 (*n* = 2) [18, 26] genotypes. All used a genomic reference standard.

**Table 4:** Characteristics of studies reporting test accuracy of ultrasound.

Author, year, country	Population	*N* in study	Index test criteria for ADPKD	Ultrasound technology	Reference standard*	Age bands reported	Gene subgroups reported
**Ultrasound**							
First-degree relatives							
Parfrey 1990 [27] (Bear 1992) Canada	1st degree family members of *PKD1* families (confirmed by gene linkage)	126 people from 10 PKD1 families	1+/2+	NR	Gene linkage analysis	</> 30 years	All were *PKD1*
Elles 1994 [24] UK	1st degree relatives of ADPKD (criteria for probands unclear)	80	Bear 1984 1+/2+	3.5-MHz scanner	Genomic markers	</> 30 years	Only reports results for *PKD1*
Ravine 1994 [28] Australia	Undiagnosed 1st degree relatives of confirmed *PKD1* probands	204 (from 18 families)	1+/2+	3- or 5-MHz	>95% or <5% probability of PKD1 by DNA linkage analysis	15–29 ≥30	All were *PKD1*
Gabow 1997 [25]	Children 1st degree relatives of ADPKD1 families (genomically confirmed)	106 children (from 40 families)	Any cysts	NR	Gene linkage analysis	Children	All were *PKD1*
Nicolau 1999 [26] Spain	1st degree relatives of Type 1 or Type 2 ADPKD (genomically confirmed)	319 individuals from 54 families	Ravine 1994: <30: 2+ in total 30–59: 2+/2+ >60: 4+/4+	3.7 or 5-MHz	Genetic linkage study	</>30 years	*PKD1* *PKD2*
Demetriou 2000 [23] Cyprus	1st degree relatives of ADPKD Type 2 families	211 alive people at risk from 3 families	Ravine 1994 (ADPKD-1) for adults and Gabow 1997 for children 5–14: 1+ Cysts 15–19: 1+/1+ Cysts Or 2+/020–29: 2+/1+ (3+ and bilateral involvement) 30–59: 2+/2+ 60>: 4+/4+	3.5 or 5-MHz	DNA linkage and direct mutation analyses	5–14 15–19 20–29 30–59 60>	All were *PKD2*
Pei 2009 [18] Australia, Europe, USA	1st degree relatives at risk of PKD1 or PKD2 (proband diagnostic criteria unclear)	948	15–39: 3+ total 40–59: 2+/2+ ≥60: 4+/4+	3- or 5-MHz	Genomic testing (range of methods)	15–29 30–39 40–59 60+	*PKD1* *PKD2* Simulated cohort of mixed PKD1/2

NR, not reported.

**Figure 5:**
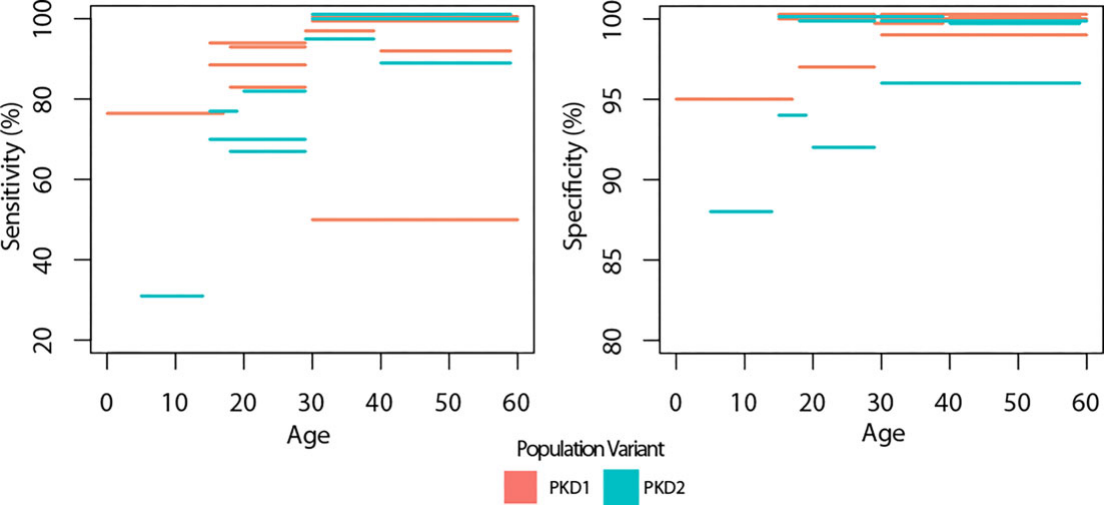
Sensitivity and specificity of ultrasound studies. Each bar represents the sensitivity or specificity for the age range spanned by the bar, as reported by individual studies included in this review.

Both sensitivity and specificity improved as age increased (see Fig. 5 and Online Supplement 4), across both *PKD1* and *PKD2* populations, but accuracy was poorer in *PKD2* compared to *PKD1* populations. The lowest sensitivity and specificity were 31% and 88%, respectively, reported in *PKD2* populations aged 5–14. The highest were 100% and 100%, respectively, in multiple gene/age categories.

## DISCUSSION

Using 58 studies of genomic (*n* = 51) and ultrasound (*n* = 7) testing spanning over 30 years, this review charts the evolving methods available for screening first-degree relatives of people affected by ADPKD. Notably, none of the genomic studies we found recruited relatives at 50% risk of ADPKD, meaning the accuracy of the tests in this population is unclear. The available evidence suggests that, among people who have a clinical diagnosis of ADPKD but they or their family have not previously had genomic testing, the sensitivity of genomic tests is likely to be somewhere between 70% and 80%, depending on test methodology and the proportion of unknown variants within the population sample. Sensitivities lower than 50% and higher than 90% have also been reported.

Due to technological advances and increased sharing of known pathogenic variants we expected to see an increase in sensitivity over time, but instead the evidence suggests that the detection rate has not changed greatly. Possible explanations for these findings include (i) the small impact that increased testing of a panel of cytogenic genes has when the vast majority of pathogenic variants are in *PKD1* and *PKD2*; (ii) pathogenic variants not detected by the methodology used e.g. deep intronic variants/structural variants/regulatory variants; (iii) unique variants detected with insufficient evidence to reach a (likely) pathogenic score (i.e. variant of uncertain significance); (iv) other cystogenic genes being responsible; (v) other causes e.g. simple age-related cysts; and (vi) the observed widening recruitment criteria leading to more atypical cases being recruited, and an increase in the size of the population for which genomic testing has become relevant, owing to the identification of additional rare ADPKD genes such as *GANAB* and *HNF1B, COL4A1, DNAJB11, REN*, and *UMOD* (see Table 3). However, only one study in this review included the recently identified *IFT140* gene that has been shown to be the third most common associated with ADPKD after *PKD1* and *PKD2* [79]. The extent and rate at which current gene panels have adopted these more recently identified variants was not the subject of our study, but it is possible laboratories may not wait for extensive publication on variants before incorporating them in their gene panels.

Meanwhile, no studies on the performance of ultrasound screening in first-degree relatives of families with the more contemporary known pathogenic variants were identified. Consequently, the test accuracy of ultrasound outside populations with *PKD1* and *PKD2* is currently unknown. The KDIGO guidelines [11] recommend that when making an initial diagnosis of ADPKD in an adult at risk, abdominal imaging by ultrasound can be used even when the family is genetically resolved. This is despite the lack of evidence in populations outside PKD1 and PKD2. Whereas genetically unresolved families are reliant on this screening modality and guidelines continue to recommend the use of ultrasound in families with other variants, further studies are required to establish the accuracy of the test in these populations. Clinicians may need to keep these uncertainties in mind when planning further monitoring and when considering alternative diagnoses.

In clinical practice, relatives of individuals who have no pathogenic variant identified by genomic testing may be receiving radiological screening tests derived and validated in populations who broadly speaking have different pathogenic variants, since our review found all ultrasound studies recruited patient with known PKD1 or PKD2. This may lead to uncertainty in clinical diagnoses, or incorrect exclusion of disease in relatives who are still in the early stages of a clinical disease with a more slowly progressing natural history.

This systematic mapping review has been conducted to the same standards as a systematic review in terms of the search methodology, study selection, and data extraction. Data were organized according to several factors that may affect test metrics, including the recruitment criteria and reference standards used. Nevertheless, it does have some methodological limitations, often generated by the available evidence. The lack of data on diagnostic test accuracy of genomic tests in people at risk of ADPKD lead to protocol amendments including widening criteria to include studies reporting only sensitivity and in people with clinically confirmed ADPKD (removing the requirement for this to be confirmed after age 40). As a result, the included studies were not true diagnostic test accuracy studies. Critical appraisal using QUADAS-2 [22] was not performed because it is not designed for these studies and would have been uninformative. Heterogeneity in populations and test methodologies precluded meta-analysis. Since the genomic studies did not specify that included patients had to have a radiological diagnosis after a certain age, and since cysts tend to increase over time, the populations recruited according to these criteria may include more patients who presented at a young age and therefore have more progressive disease. Finally, there will inevitably remain some heterogeneity in how the pathogenic categories were defined, especially as new variants were identified and guidelines to determine variant pathogenicity have changed over time [80].

Policy makers should consider the generalisability of the patient populations recruited to the studies, which are broadening over time, to their own populations. The specifics of the test methodologies with respect to available expertise, equipment, and small incremental gains of the technologies and additional variants should also be considered.

In conclusion, this study demonstrates that while genomic testing methods have advanced, detection rates have not greatly improved, possibly due to wider inclusion criteria, and the small incremental gains of testing genes other than PKD1 and PKD2. For people at risk of ADPKD in genetically unresolved families, the accuracy of ultrasound is uncertain, and clinical communities should bear this in mind when screening for ADPKD.

## Supplementary Material

sfaf187_Supplemental_Files

## Data Availability

Data underpinning this review are available from the authors on request.
