# Supplementary material for: The diagnostic accuracy of ultrasound and genomic tests for the diagnosis of autosomal-dominant polycystic kidney disease: a systematic mapping review
Source: Clin Kidney J. 2025 Jun 13;18(7):sfaf187. doi: 10.1093/ckj/sfaf187 (PMC12280278; doi:10.1093/ckj/sfaf187)
Supplement: sfaf187_Supplemental_Files [file sfaf187_supplemental_files.zip › Supplement 1 Protocol amendments.docx]

Protocol changes

The protocol was registered on the PROSPERO database (record number CRD42023456727), but some changes were made to the protocol. We widened our inclusion criteria to include studies that were not true diagnostic test accuracy studies, and to redefine the reference standards required (further details are given in *Study Selection* below). This was because there were no studies meeting the predefined criteria. We initially planned a full systematic review, however, it became evident that the nature of the evidence base was more suited to a systematic mapping review because of the rapidly evolving and highly heterogeneous nature of the genetic tests, because the available studies were not true diagnostic test accuracy studies and could not be adequately assessed using existing quality assessment tools (e.g. QUADAS 2), and because there was a large volume of this poorer quality literature. The mapping review format allowed us to focus on characterising the existing genetic tests and chart their evolution over time, whilst still allowing the presentation of the available accuracy metrics (detection rate).

The reference standard criteria for genetic studies could be a diagnosis using published criteria, or a genetic diagnosis. This was a change from the published protocol, which specified that the reference standard should be ultrasound after age 40 years. We widened the criterion since no studies met the original criterion.

The criteria relating to the population was widened in accordance with the protocol to include studies in people with clinically confirmed ADPKD, rather than just those at 50% risk.
