# Supplementary material for: The diagnostic accuracy of ultrasound and genomic tests for the diagnosis of autosomal-dominant polycystic kidney disease: a systematic mapping review
Source: Clin Kidney J. 2025 Jun 13;18(7):sfaf187. doi: 10.1093/ckj/sfaf187 (PMC12280278; doi:10.1093/ckj/sfaf187)
Supplement: sfaf187_Supplemental_Files [file sfaf187_supplemental_files.zip › Supplement 2 Additional info review methodology.docx]

**Online supplement 2**

**This supplement contains additional information about the review methods.**

1. **Search strategies – pg 2**
2. **Data extraction – pg 5**
3. **Search strategies**

All structured database searches were run on August 18^th^ and 20^th^, 2023. The additional searching was conducted between September and October, 2023.

**MEDLINE (Ovid)** and Ovid MEDLINE(R) In-Process & Other Non-Indexed Citations

1946 to August 18, 2023

**1**  Polycystic Kidney Diseases/
**2**  Polycystic Kidney, Autosomal Dominant/
**3**  polycystic kidney disease*.tw.
**4**  ADPKD.tw.
**5**  (PKD or PKD1 or PKD2).tw.
**6**  or/1-5
**7**  exp ultrasonography/
**8**  (ultrasonic adj (diagnos* or tomograph* or imaging*)).tw.
**9**  ultrasonograph*.tw.
**10**  ultrasound.tw.
**11**  diagnostic imaging.fs.
**12**  exp Genetic Testing/
**13**  exp Whole Genome Sequencing/
**14**  exp High-Throughput Nucleotide Sequencing/
**15**  (gene* adj3 (test* or screen*)).tw.
**16**  ((genome or next generation or exome) adj2 sequenc*).tw.
**17**  or/7-16 (2139695)
**18**  sensitiv*.mp.
**19**  predictive value*.mp.
**20**  accurac*.tw.
**21**  or/18-20
**22**  and/6,17,21

**Embase (Ovid)** 1974 to 2023, Week 33

**1**  kidney polycystic disease/
**2**  polycystic kidney disease*.tw.
**3**  ADPKD.tw.
**4**  (PKD or PKD1 or PKD2).tw.
**5**  or/1-4
**6**  exp echography/
**7**  (ultrasonic adj (diagnos* or tomograph* or imaging*)).tw.
**8**  ultrasonograph*.tw.
**9**  ultrasound.tw.
**10**  exp diagnostic imaging/
**11**  exp genetic screening/
**12**  exp whole genome sequencing/
**13**  exp high throughput sequencing/
**14**  (gene* adj3 (test* or screen*)).tw.
**15**  ((genome or next generation or exome) adj2 sequenc*).tw.
**16**  or/6-15
**17**  sensitiv:.tw.
**18**  diagnostic accuracy.sh.
**19**  diagnostic.tw.
**20**  or/17-19
**21**  and/5,16,20

**The Cochrane Library** (John Wiley & Sons, Ltd)

#1 MeSH descriptor: [Polycystic Kidney Diseases] this term only

#2 MeSH descriptor: [Polycystic Kidney, Autosomal Dominant] this term only

#3 polycystic kidney disease*:ti,ab

#4 ADPKD:ti,ab

#5 (PKD or PKD1 or PKD2):ti,ab

#6 #1 OR #2 OR #3 OR #4 OR #5

#7 MeSH descriptor: [Ultrasonography] explode all trees

#8 (ultrasonic adj (diagnos* or tomograph* or imaging*)):ti,ab

#9 ultrasonograph*:ti,ab

#10 ultrasound:ti,ab

#11 MeSH descriptor: [] explode all trees and with qualifier(s): [diagnostic imaging - DG]

#12 MeSH descriptor: [Genetic Testing] explode all trees

#13 MeSH descriptor: [Whole Genome Sequencing] explode all trees

#14 MeSH descriptor: [High-Throughput Nucleotide Sequencing] explode all trees

#15 (gene* NEAR/3 (test* or screen*)):ti,ab

#16 ((genome or "next generation" or exome) NEAR/2 sequenc*):ti,ab

#17 #7 OR #8 OR #9 OR #10 OR #11 OR #12 OR #13 OR #14 OR #15 OR #16

#18 #6 AND #17

**Conferences searched for relevant abstracts**

American Society of Nephrology Kidney Week

World Congress of Nephrology

European Renal Association Congress

**List of articles used for PubMed Similar Article searching, Web of Science reference lists and cited reference searching**

**Genetic:**

**#1435 - Mantovani 2020** - Gene Panel Analysis in a Large Cohort of Patients With Autosomal Dominant Polycystic Kidney Disease Allows the Identification of 80 Potentially Causative Novel Variants and the Characterization of a Complex Genetic Architecture in a Subset of Families
Mantovani, V.; Bin, S.; Graziano, C.; Capelli, I.; Minardi, R.; Aiello, V.; Ambrosini, E.; Cristalli, C. P.; Mattiaccio, A.; Pariali, M.; De Fanti, S.; Faletra, F.; Grosso, E.; Cantone, R.; Mancini, E.; Mencarelli, F.; Pasini, A.; Wischmeijer, A.; Sciascia, N.; Seri, M.; La Manna, G.
Frontiers in Genetics 2020;11 (no pagination)():
DOI: 10.3389/fgene.2020.00464 · Ref ID: 796

**#1282 - Elles 1994 -** Diagnosis of adult polycystic kidney disease by genetic markers and ultrasonographic imaging in a voluntary family register
Elles, R. G.; Hodgkinson, K. A.; Mallick, N. P.; O'Donoghue, D. J.; Read, A. P.; Rimmer, S.; Watters, E. A.; Harris, R.
J Med Genet 1994;31(2):115-20

**#1457 - Mallawaarachchi 2021** - Genomic diagnostics in polycystic kidney disease: an assessment of real-world use of whole-genome sequencing
Mallawaarachchi, A. C.; Lundie, B.; Hort, Y.; Schonrock, N.; Senum, S. R.; Gayevskiy, V.; Minoche, A. E.; Hollway, G.; Ohnesorg, T.; Hinchcliffe, M.; Patel, C.; Tchan, M.; Mallett, A.; Dinger, M. E.; Rangan, G.; Cowley, M. J.; Harris, P. C.; Burnett, L.; Shine, J.; Furlong, T. J.
Eur J Hum Genet 2021;29(5):760-770
DOI: 10.1038/s41431-020-00796-4

**#1223 - Hu 2021-** Comprehensive strategy improves the genetic diagnosis of different polycystic kidney diseases
Hu, H. Y.; Zhang, J.; Qiu, W.; Liang, C.; Li, C. X.; Wei, T. Y.; Feng, Z. K.; Guo, Q.; Yang, K.; Liu, Z. G.

Journal of Cellular and Molecular Medicine 2021;25(13)():6318-6332
DOI: 10.1111/jcmm.16608

**#1661 - Tan 2009** - Novel method for genomic analysis of PKD1 and PKD2 mutations in autosomal dominant polycystic kidney disease
Tan, Y. C.; Blumenfeld, J. D.; Anghel, R.; Donahue, S.; Belenkaya, R.; Balina, M.; Parker, T.; Levine, D.; Leonard, D. G.; Rennert, H. Hum Mutat 2009;30(2):264-73 DOI: 10.1002/humu.20842

**Ultrasound**

**#1949 - Pei 2009** - Unified criteria for ultrasonographic diagnosis of ADPKD. Pei, Y.; Obaji, J.; Dupuis, A.; Paterson, A. D.; Magistroni, R.; Dicks, E.; Parfrey, P.; Cramer, B.; Coto, E.; Torra, R.; San Millan, J. L.; Gibson, R.; Breuning, M.; Peters, D.; Ravine, D. J Am Soc Nephrol 2009;20(1):205-12
DOI: 10.1681/ASN.2008050507

1. **Data extraction fields**

Data extraction fields included population recruitment criteria, family history, number in analysis, country of recruitment, reference standard, genes targeted, test methods, name of sequencer, detection rate and numbers in the different pathogenicity categories. After data extraction, some fields (population; reference standard; test type) were re-coded to simplify the process of mapping studies. All data and codes were then checked by a second reviewer and differences resolved through discussion, or through the involvement of a third reviewer.
