# Supplementary material for: The diagnostic accuracy of ultrasound and genomic tests for the diagnosis of autosomal-dominant polycystic kidney disease: a systematic mapping review
Source: Clin Kidney J. 2025 Jun 13;18(7):sfaf187. doi: 10.1093/ckj/sfaf187 (PMC12280278; doi:10.1093/ckj/sfaf187)
Supplement: sfaf187_Supplemental_Files [file sfaf187_supplemental_files.zip › Supplement 4 Ultrasound data table.docx]

| **Study ID** | **Sensitivity** | | | | | | | | | | | **Specificity** | | | | | | | | | | |
| --- | --- | --- | --- | --- | --- | --- | --- | --- | --- | --- | --- | --- | --- | --- | --- | --- | --- | --- | --- | --- | --- | --- |
|  | <18 | <30 | >30 | 5-14 | 15-19 | 20-29 | 30-59 | 15-29 | 30-39 | 40-59 | 60+ | <18 | <30 | >30 | 5-14 | 15-19 | 20-29 | 30-59 | 15-29 | 30-39 | 40-59 | 60+ |
| **PKD1** | | | | | | | | | | | | | | | | | | | | | | |
| Elles 1994 |  | 93 (77, 99) | 50 (1, 99) |  |  |  |  |  |  |  |  |  | 97 (82, 100) | 100 (83, 100) |  |  |  |  |  |  |  |  |
| Ravine 1994 |  |  | 100 (75, 100) |  |  |  |  | 88.5 (NR) |  |  |  |  |  | 99 (94, 100) |  |  |  |  | 100 (92, 100) |  |  |  |
| Nicolau 1999 |  | 95 (88, 99) | 100 (96, 100) |  |  |  |  |  |  |  |  |  | 100 (94, 100) | 100 (91, 100) |  |  |  |  |  |  |  |  |
| Parfrey 1990 |  | 83 (70, 93) | 100 (87, 100 |  |  |  |  |  |  |  |  |  | 100 (85, 100) | 100 (88, 100) |  |  |  |  |  |  |  |  |
| Gabow 1997 | 77 (65, 87) |  |  |  |  |  |  |  |  |  |  | 95 (85, 99) |  |  |  |  |  |  |  |  |  |  |
| Pei 2009 |  |  |  |  |  |  |  | 94 (89, 98) | 97 (91, 100) | 92 (83, 100) | 100 |  |  |  |  |  |  |  | 100 | 100 | 100 |  |
| **PKD2** | | | | | | | | | | | | | | | | | | | | | | |
| Nicolau 1999 |  | 67 (22, 96) | 100 (77, 100) |  |  |  |  |  |  |  |  |  | 100 (66, 100) | 100 (48, 100) |  |  |  |  |  |  |  |  |
| Demetriou 2000 |  |  |  | 31 (11, 59) | 77 (46, 95) | 82 (63, 94) | 100 (93, 100) |  |  |  |  |  |  |  | 88 (62, 98) | 94 (73, 100) | 92 (75, 99) | 96 (85, 99) |  |  |  |  |
| Pei 2009 |  |  |  |  |  |  |  | 70 (57, 81) | 95 (88, 100) | 89 (77, 97) | 100 |  |  |  |  |  |  |  | 100 | 100 | 100 |  |
| **Simulation of accuracy in mixed PKD1 and PKD2 cohort** | | | | | | | | | | | | | | | | | | | | | | |
| Pei 2009 |  |  |  |  |  |  |  | 82 (75, 88) | 96 (92, 99) | 90 (83, 96) | 100 |  |  |  |  |  |  |  | 100 | 100 | 100 |  |
