# Supplementary figures and images for: The diagnostic accuracy of ultrasound and genomic tests for the diagnosis of autosomal-dominant polycystic kidney disease: a systematic mapping review
Source: Clin Kidney J. 2025 Jun 13;18(7):sfaf187. doi: 10.1093/ckj/sfaf187 (PMC12280278; doi:10.1093/ckj/sfaf187)

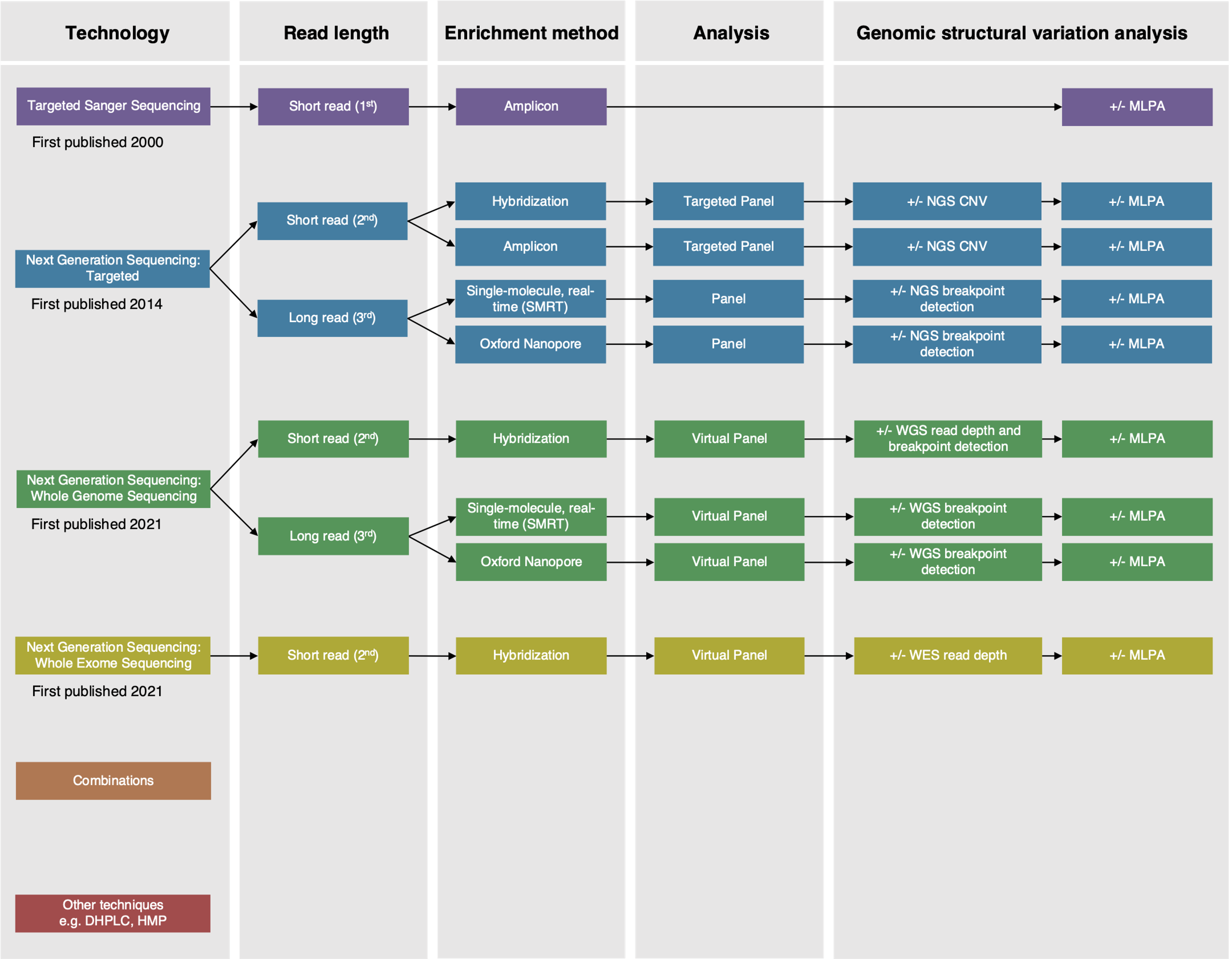

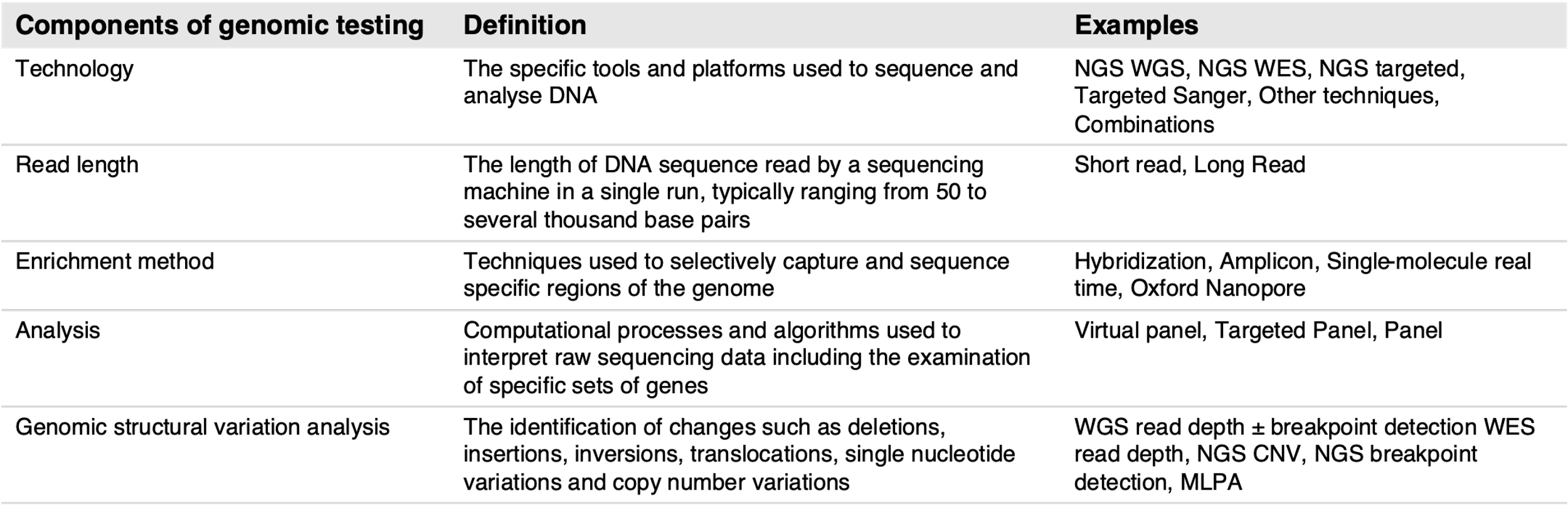

Supplement: sfaf187_Supplemental_Files [file sfaf187_supplemental_files.zip › Supplement 3 Genomic test taxonomy.docx]
